# ROS-Responsive Micelles Loaded with Podophyllotoxin Inhibit Tumor Growth via ROS Self-Amplification and Regulation of Survivin and p21 Expression

**DOI:** 10.3390/ijms27156546

**Published:** 2026-07-23

**Authors:** Shuaiheng Song, Qiang Shao, Siyi Liang, Haoyang Du, Qingnan Zhao, Jing Guan, Ping Lin, Feng Lin

**Affiliations:** 1 Department of Organic Chemistry, College of Pharmacy, Harbin Medical University, Harbin 150081, China; 2022020353@hrbmu.edu.cn (S.S.); sq17346730606@126.com (Q.S.); siyi20242024@163.com (S.L.); du8462795130@163.com (H.D.); zhaoqn@hrbmu.edu.cn (Q.Z.); guanjing@ems.hrbmu.edu.cn (J.G.); 2 Department of Biochemistry and Molecular Biology, College of Basic Medical Science, Harbin Medical University, Harbin 150081, China

**Keywords:** micelles, ROS response, podophyllotoxin, cinnamaldehyde, thioacetal

## Abstract

Podophyllotoxin (PPT) inhibits tumors such as lung cancer and breast cancer. However, it has poor water solubility and causes gastrointestinal dysfunction and bone marrow suppression, which severely limit its clinical application. Based on the differential reactive oxygen species (ROS) levels between tumor microenvironments and normal tissues, we designed and constructed a ROS-responsive micelle delivery system, successfully fabricating blank micelles (M) and PPT-loaded micelles (M@PPT). Both micelles exhibited good particle size uniformity, colloidal stability, and biosafety. The ROS responsiveness experiment revealed that, after incubating blank micelles (M) with 10 mM H_2_O_2_, the particle size increased significantly, and the size distribution broadened. High-performance liquid chromatography (HPLC) confirmed the release of cinnamaldehyde from the micelles upon H_2_O_2_ exposure. Additionally, DCFH-DA assays demonstrated that treatment with blank micelles (M) enhanced intracellular ROS levels. In vitro release studies showed that drug-loaded micelles (M@PPT) achieved 77.76% cumulative PPT release within 24 h in a buffer containing 10 mM H_2_O_2_, significantly exceeding the release observed in the H_2_O_2_-free control group. These results collectively validate the ROS-responsive disintegration of micelles and the subsequent release of cinnamaldehyde. The liberated cinnamaldehyde further amplified intracellular ROS levels, establishing a positive feedback loop that accelerated drug release. Cellular assays revealed superior tumor growth inhibition by M@PPT over free PPT, mediated through apoptosis induction, G2/M phase cell cycle arrest, downregulation of the anti-apoptotic protein Survivin, and upregulation of the p21 protein. In vivo studies further confirmed the enhanced antitumor efficacy and improved biosafety of M@PPT compared to free PPT. This ROS-responsive micellar system, by enabling tumor-targeted drug delivery and controlled release, provides a novel strategy to optimize the clinical utility of podophyllotoxin-based chemotherapeutics.

## 1. Introduction

Nowadays, the incidence and mortality of malignant tumors keep rising, making cancer a severe threat to human health [1]. Clinically, tumor treatments mainly include surgical resection, chemotherapy, and radiotherapy [2]. Chemotherapy has an irreplaceable therapeutic effect on advanced tumors and postoperative adjuvant intervention by killing proliferative tumor cells [3]. However, conventional small-molecule chemotherapeutics suffer from prominent drawbacks: poor water solubility, lack of tumor selectivity, severe systemic toxicities against normal tissues, and long-term induced drug resistance [4,5]. These drawbacks substantially restrict their clinical translational potential.

Nanoparticle drug delivery systems have been developed to address the above limitations. Drugs can be encapsulated inside nanocarriers via physical embedding or covalent conjugation and passively accumulate at tumor lesions, relying on the tumor enhanced permeability and retention (EPR) effect, thereby elevating intratumoral drug accumulation [6,7]. Common nanocarriers include liposomes [8], polymeric micelles [9], clathrate compounds [10], and inorganic metal nanoparticles [11]. Among them, liposomes and micelles have mature preparation techniques, and multiple formulations have entered late clinical trials or clinical practice in some countries [12]. Nevertheless, the EPR effect shows obvious individual differences in human solid tumors, due to heterogeneous vascular structures and tissue-specific characteristics of human tumor microenvironments [13,14]. More importantly, traditional non-responsive nanocarriers cannot precisely control drug release. Drugs tend to leak prematurely in the circulation, so they not only cause serious off-target damage to healthy organs, but also fail to reach effective therapeutic concentrations at tumor sites.

To overcome premature leakage and insufficient tumor targeting, tumor microenvironment-activated stimulus-responsive nanocarriers have become a popular research direction. Representative responsive designs are based on weak tumor acidity [15], elevated reactive oxygen species (ROS) [16], high intracellular glutathione (GSH) [17], and tumor-specific enzymes [18,19]. These intelligent carriers maintain stable structures under normal physiological conditions and disintegrate to release drugs only when exposed to tumor-specific stimuli, which effectively improves targeting efficiency and reduces systemic side effects [20].

ROS-responsive nanoplatforms attract widespread attention because tumor cells possess abnormally vigorous oxidative metabolism and maintain far higher ROS levels than normal cells [21]. Conventional single ROS-responsive nanocarriers have unique advantages: they can respond to the endogenous high ROS levels in tumors to trigger carrier degradation and localized drug release, achieving better tumor selectivity than non-responsive nano-formulations. Even so, single ROS-sensitive systems still have several critical unresolved challenges that restrict their antitumor efficacy. First, single ROS-cleavable linkages are only sensitive to high ROS inside tumor cores, showing weak responsiveness at tumor margins with low ROS, leading to incomplete drug release and high recurrence risk. Second, low physiological ROS in normal tissues may induce slight non-specific cleavage of responsive bonds, resulting in unwanted drug leakage and residual toxicity. Third, single responsive units cannot amplify intracellular ROS after degradation, lacking a positive feedback cascade that facilitates progressive carrier degradation. To address these bottlenecks, researchers have constructed dual ROS-responsive nanocarriers with synergistic multi-stage response mechanisms. Hou et al. reported hyaluronic acid–ferrocene dual ROS-responsive micelles (HASF) containing thioacetal linkages (TK); ROS-triggered ferrocene oxidation changes carrier hydrophobicity and disintegrates micelles to boost curcumin delivery [22]. Meanwhile, Xu et al. fabricated pH/ROS dual-sensitive polymeric prodrug nanoparticles (PVD-NPs) based on PEG-b-PLL copolymers, combining TK-conjugated paclitaxel prodrugs and charge-reversal DMA groups to enhance cellular uptake and ROS-accelerated drug release [23]. These dual ROS designs verify the superiority of multi-level ROS-activated delivery, yet most reported dual ROS nano-systems suffer from rapid in vivo clearance and weak tumor enrichment capacity.

Polyethylene glycol (PEG) modification is a classic and necessary optimization strategy matched with ROS-responsive nanocarriers, solving the critical circulation defect of most ROS-sensitive polymers. The hydrophilic PEG chain forms a hydration layer on micelle surfaces through steric hindrance, repelling plasma protein adsorption and avoiding rapid elimination by the reticuloendothelial system (RES), thereby significantly prolonging blood circulation time [24]. Without PEG decoration, most ROS-responsive micelles are easily captured by macrophages and fail to deliver enough drug to tumor sites. However, ordinary PEGylated single ROS nanocarriers still cannot dynamically adjust drug release according to the gradient ROS distribution in tumors, which inspires us to combine PEG long-circulation modification with self-amplified ROS cascade-responsive structures to balance stable circulation and tumor-selective rapid drug release.

Podophyllotoxin (PPT) is selected as the therapeutic payload of our system because of its outstanding antitumor potential. As a natural aryl-tetralin lignan, PPT strongly suppresses the proliferation of multiple tumor types by inhibiting tubulin polymerization, disrupting mitotic spindle formation, arresting the cell cycle at the G2/M phase, and inducing tumor cell apoptosis [25,26]. Unfortunately, free PPT faces multiple translational barriers: poor water solubility leads to fast metabolic clearance; non-specific distribution causes severe toxicity against proliferative normal tissues, including bone marrow and tissues in the gastrointestinal tract; and rapid degradation in the circulation cannot sustain effective tumoricidal concentrations inside tumors [27,28]. Traditional PEG-modified non-responsive nanocarriers merely improve PPT water solubility but cannot prevent premature drug leakage in healthy tissues or realize tumor-specific triggered release, resulting in limited therapeutic efficacy and safety risks. Benefiting from the distinct ROS concentration gap between tumor and normal tissues, PEG-functionalized ROS-responsive micelles are ideal carriers to overcome the clinical transformation obstacles of free PPT.

**Figure 1:**
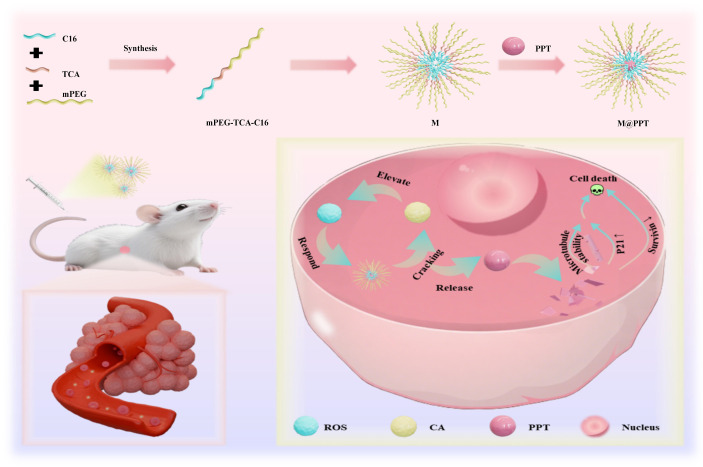
Schematic diagram of reactive cleavage and PPT release of ROS-responsive drug-loaded nano-micelles (M@PPT).

In this study, we designed and constructed a thioacetal bond-based ROS self-amplified nano-delivery system M@PPT. We synthesized an amphiphilic copolymer (mPEG-TCA-C16) by connecting methoxy polyethylene glycol (mPEG2000) with hexadecyl-modified thioacetal-cinnamaldehyde intermediate (TCA), which self-assembles into blank micelles (M) in an aqueous environment. The hexadecyl-conjugated thioacetal-cinnamaldehyde (TCA-C16) segment acts as the hydrophobic core unit carrying cleavable ROS-sensitive thioacetal bonds, while the mPEG segment forms the hydrophilic outer shell to guarantee a long blood circulation time. After encapsulating PPT, drug-loaded micelles (M@PPT) were obtained. We hypothesize that the thioacetal bonds will be cleaved by the high ROS levels within tumor microenvironments to release cinnamaldehyde. The liberated cinnamaldehyde consumes intracellular reducing substances and further elevates ROS levels, forming a positive feedback loop to accelerate micelle disassembly and PPT release (Figure 1). The mPEG shell ensures systemic circulation stability, while the ROS-responsive TCA segment endows micelles with tumor-specific intelligent drug release performance.

## 2. Results

### 2.1. Synthesis of mPEG-TCA-C16

The complete synthetic pathway for the thioacetal-cinnamaldehyde intermediate (TCA), the hexadecyl-modified thioacetal-cinnamaldehyde intermediate (TCA-C_16_), and methoxy polyethylene glycol-TCA-C_16_ (mPEG-TCA-C_16_) is illustrated in Figure S1.

TCA was synthesized from 3-mercaptopropionic acid and cinnamaldehyde, catalyzed by trifluoroacetic acid. Then, using 1-(3-dimethylaminopropyl)-3-ethylcarbodiimide hydrochloride (EDCI) and 4-dimethylaminopyridine (DMAP) as catalysts, TCA was esterified with hexadecanol to obtain TCA-C16. Following esterification of TCA-C16 with methoxy polyethylene glycol (mPEG2000), the white powder mPEG-TCA-C16 was obtained, with a total yield of 35.2%.

### 2.2. Characterization of mPEG-TCA-C16

#### 2.2.1. FT-IR Spectra

**Figure 2:**
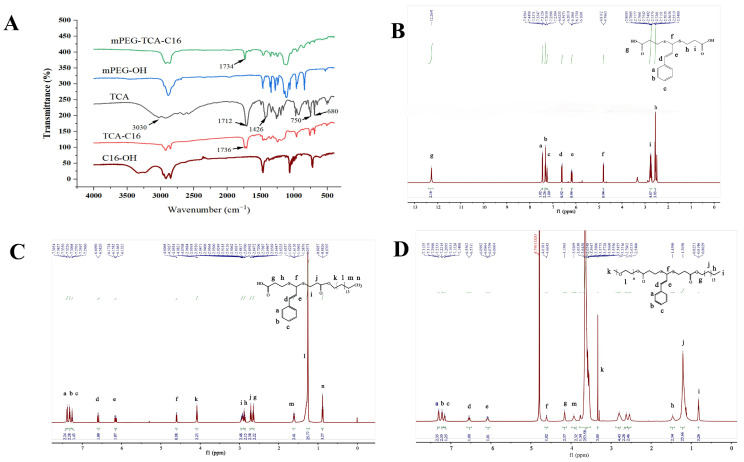
Structural characterization of mPEG-TCA. (**A**) FT-IR spectra of TCA, C16-OH, TCA-C16, mPEG-OH, and mPEG-TCA-C16. (**B**) ^1^H-NMR spectrum of TCA. (**C**) ^1^H-NMR spectrum of TCA-C16. (**D**) ^1^H-NMR spectrum of mPEG-TCA-C16.

TCA, TCA-C16, and mPEG-TCA-C16 were characterized by Fourier infrared (FI-TR) spectroscopy and proton nuclear magnetic resonance (^1^H-NMR) spectroscopy. In the infrared spectrum (Figure 2A), TCA exhibited a broad O–H stretching vibration band at 3400–2500 cm^−1^, characteristic of carboxylic acid groups with hydrogen bonding interactions, along with a C=O stretching vibration peak at 1710 cm^−1^. Aromatic C-H stretching vibrations were observed at 3030 cm^−1^, while C=C skeletal vibrations of the benzene ring appeared at 1426 cm^−1^. The γ (C–H) bending vibrations of the aromatic ring were identified at 750 cm^−1^ and 680 cm^−1^. In TCA-C16, a new ester carbonyl C=O stretching vibration emerged at 1730 cm^−1^. Comparative analysis between mPEG-TCA-C16 and mPEG-OH confirmed successful synthesis, evidenced by the appearance of an ester carbonyl C=O stretching vibration at 1734 cm^−1^ in the copolymer.

#### 2.2.2. ^1^H-NMR Spectra

In the ^1^H nuclear magnetic resonance (^1^H-NMR) spectrum for the thioacetal intermediate (TCA) (Figure 2B); the resonance at δ 7.2–7.5 ppm corresponds to aromatic protons a, b, and c, which are labeled on the benzene ring of the molecular structural scheme inset in Figure 2B. The signals at δ 6.2 ppm and δ 6.6 ppm are assigned to olefinic protons d and e marked on the C=C double bond of the same structural drawing, while the peak at δ 4.8 ppm originated from proton f located on the methyl group substituted between the two sulfur atoms in the illustrated structure. The above spectral data confirm successful synthesis of the thioacetal intermediate TCA.

In the ^1^H-NMR spectrum for TCA-C_16_ (Figure 2C), the signal at δ 0.9 ppm belongs to proton n, which labels the terminal methyl group derived from hexadecanol in the inset molecular structure. The resonance at δ 4.1 ppm corresponds to proton k at the α-hydroxyl position of hexadecanol, clearly marked on the alkyl ester segment in the structural diagram in Figure 2C. Combined with the characteristic signals observed in the ^1^H-NMR spectrum for TCA, these results verify successful synthesis of TCA-C_16_.

In the ^1^H-NMR spectrum for mPEG-TCA-C_16_ (Figure 2D), the broad signal covering δ 3.4–3.8 ppm arises from proton l assigned to the repeating ethylene glycol units (-OCH_2_CH_2_-) of the methoxy poly (ethylene glycol) chain, as labeled on the polymer backbone in the inset structural scheme. The characteristic peak at δ 0.9 ppm corresponds to the terminal methyl proton i on the hexadecyl alkyl chain, as labeled in the molecular inset in Figure 2D. In contrast, the resonance at δ 3.2 ppm originates from the methoxy group of the mPEG backbone. Resonances ranging from δ 6.0 to 7.4 ppm are attributed to aromatic protons a, b, and c and olefinic protons d and e, all labeled on the cinnamaldehyde moiety in the structural diagram. These characteristic signals collectively indicate successful synthesis of the mPEG-TCA-C_16_ copolymer.

### 2.3. Characterization and Quality Evaluation of Drug-Loaded Micelles (M@PPT) and Blank Micelles (M)

#### 2.3.1. Size, Zeta Potential, and Stability of Micelles

**Figure 3:**
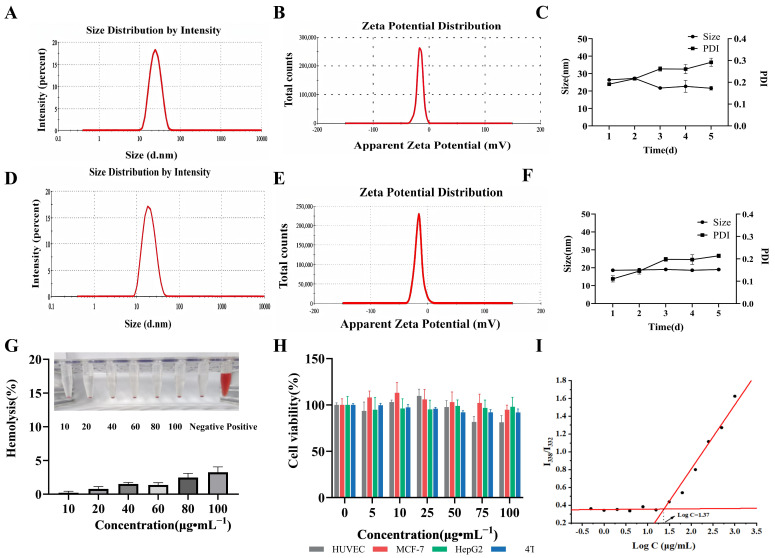
Characterization and quality evaluation of drug-loaded micelles (M@PPT) and blank micelles (M). Blank micelle (M) (**A**–**C**) and drug micelle (M@PPT) (**D**–**F**) appearance, particle size, *zeta* potential, and stability. Hemolysis (**G**) and safety (**H**) of blank micelles (M). (**I**) Critical micelle concentration of mPEG-TCA-C16 polymer.

The particle size of drug-loaded micelles (M@PPT; 18.52 ± 0.12 nm) was significantly smaller than that of blank micelles (M; 26.58 ± 0.39 nm), with polydispersity indices (PDI) of 0.144 ± 0.01 and 0.189 ± 0.07, respectively (Figure 3A,D). Small micelles (<200 nm) reduce uptake by the reticuloendothelial system and renal clearance, facilitating passive tumor accumulation via the EPR effect [29,30]. The size difference between M@PPT and M arises from hydrophobic interactions between PPT and the long alkyl chains of hexadecanol, which leads to compression of micelle core and thus reduces the particle size of drug-loaded micelles.

The *Zeta* potential of drug-loaded micelles (M@PPT) is −16.22 ± 1.85 mV, and that of blank micelles (M) is −14.9 ± 1.01 mV (Figure 3B,E). The negatively charged micellar surface repels plasma protein adsorption to prolong systemic circulation [31,32]. In addition, the encapsulation rate of M@PPT was 91.39 ± 1.06%, and the drug load was 8.31 ± 0.10%. Its high drug load could effectively improve antitumor efficacy. The specificity of the high-performance liquid chromatography (HPLC) method for podophyllotoxin (PPT) quantification was validated, and the chromatograms are displayed in Figure S2.

Both drug-loaded micelles (M@PPT) and blank micelles (M) maintained stability for 5 days at room temperature, with no significant increase in size (PDI < 0.3) (Figure 3C,F). Drug-loaded micelles (M@PPT) showed better stability, smaller particle size fluctuation range, and narrower particle size distribution.

#### 2.3.2. Hemolysis, Safety, and Critical Micelle Concentration of Blank Micelles (M)

In the biosafety evaluation, after blank micelles (M) at concentrations of 0–100 μg/mL were applied to HUVEC, MCF-7, HepG2, and 4T1 cells for 2 days, the cell survival rates were all above 80% (Figure 3H). The hemolysis experiment showed that, when the blank micelles (M) were co-incubated with red blood cells for 3 h within the concentration range of 10–100 μg/mL, there was no obvious hemolysis phenomenon in the centrifuged supernatant, and the hemolysis rate was lower than <5% (Figure 3G). These results indicate that micelles (M) possess low cytotoxicity and good biocompatibility, meeting the biosafety requirements for nanomedicine carriers.

In addition, the critical micelle concentration (CMC) of blank micelles (M) was determined to be 23.44 μg/mL using a pyrene-based fluorescence probe method (Figure 3I). This CMC value demonstrates its excellent structural stability in vivo, thereby minimizing drug leakage [33].

#### 2.3.3. Cell Uptake of Blank Micelles (M)

**Figure 4:**
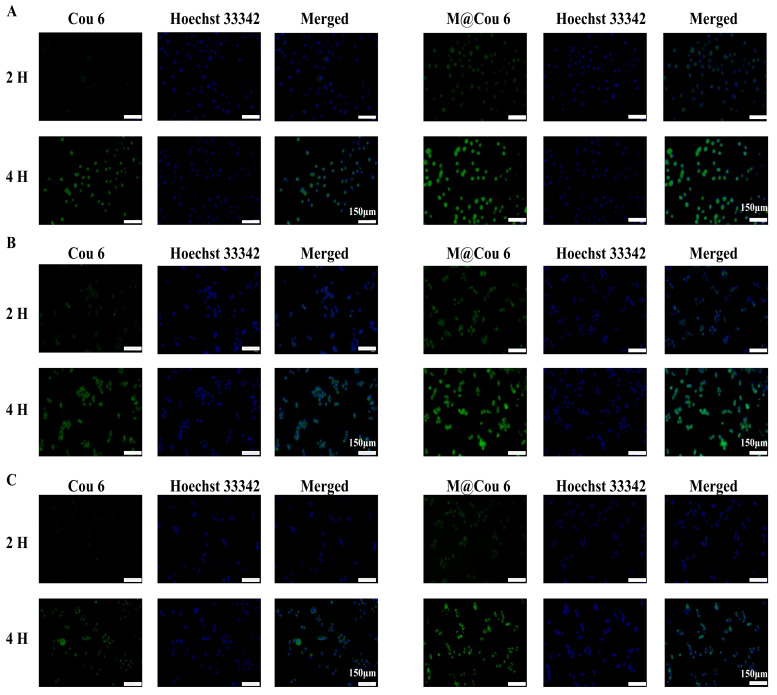
Uptake of Cou6-loaded micelles (M@Cou6) by MCF-7 (**A**), HepG2 (**B**), and 4T1 (**C**) cells.

In cell uptake experiments (Figure 4), green fluorescence was observed in MCF-7, HepG2, and 4T1 cells treated with both coumarin 6-loaded micelles (M@Cou6) and free Cou6 at 2 h and 4 h, with fluorescence intensity progressively increasing over time. Notably, the M@Cou6 group exhibited stronger fluorescence signals than the free Cou6 group at both time points, suggesting enhanced cellular uptake of M@Cou6 compared to free Cou6. This disparity originates from the divergent cellular uptake pathways of the two formulations. Free Cou6 primarily enters cells via passive diffusion, a process constrained by its lipid–water partition coefficient and the saturation limits of the carrier proteins. In contrast, the nanosized micellar M@Cou6 achieves efficient cellular internalization through endocytosis, thereby circumventing the inherent limitations of passive diffusion-mediated transport [34]. The quantitative fluorescence intensity data for coumarin 6 (Cou6)-loaded M micelles at 2 h and 4 h are summarized in Figure S3.

### 2.4. ROS Response of Drug-Loaded Micelles (M@PPT) and Blank Micelles (M)

#### 2.4.1. ROS Response of Blank Micelles (M)

**Figure 5:**
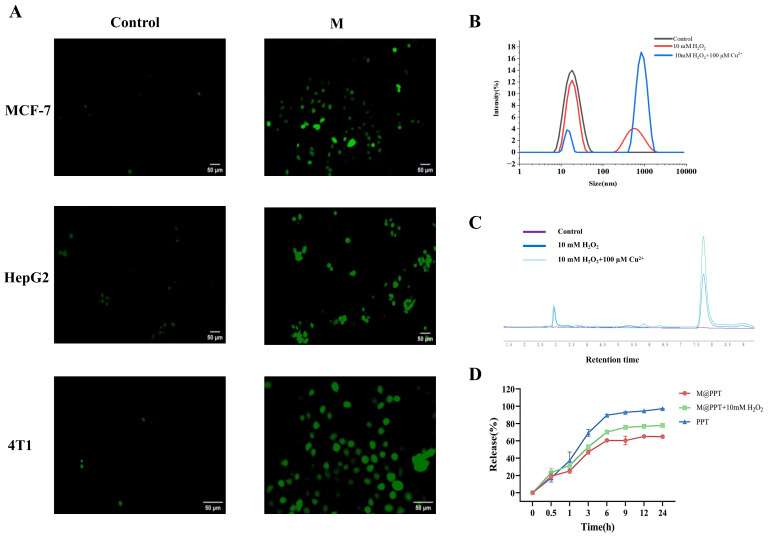
Analysis of the ROS responsiveness of the drug-loaded micelles (M@PPT) and the blank micelles (M). (**A**) Detection of the effect of the blank micelles (M) on intracellular ROS using a DCFH-DA probe. (**B**) Particle size distribution of the blank micelles (M) after incubation with 10 mM H_2_O_2_ or a Fenton-like reagent (10 mM H_2_O_2_ + 100 μM Cu^2+^) for 12 h. (**C**) The HPLC spectrum of the incubation solution. (**D**) The in vitro release results of the drug-loaded micelles (M@PPT) and free PPT.

For the ROS response experiments (Figure 5B), blank micelles (M) were incubated with 10 mM H_2_O_2_ or with a Fenton-like reagent (10 mM H_2_O_2_ + 100 μM Cu^2+^) for 12 h. The untreated blank micelles (M) showed a single-peak distribution, while the particle size distribution of the H_2_O_2_-treated group changed from a single peak to a double peak and became significantly broader. The particle size distribution of the Fenton-like reagent group was further expanded, mainly concentrated around 1000 nm. This indicates that the blank micelles (M) are ROS-responsive and dissociate under ROS stimulation, as well as being more sensitive to the hydroxyl radicals (•OH) generated by the Fenton-like reagent.

The incubation solutions described above were analyzed by HPLC (Figure 5C). No cinnamaldehyde chromatographic peak was detected in the control group, but there were obvious cinnamaldehyde chromatographic peaks in both the H_2_O_2_ and Fenton-like reagent groups, and the chromatographic peak area of the Fenton-like reagent group was larger. These results confirm that the thioacetal bonds in blank micelles (M) undergo ROS-triggered cleavage, leading to cinnamaldehyde liberation under oxidative stress conditions.

#### 2.4.2. Intracellular ROS Generation

Intracellular ROS levels were detected with the fluorescent probe DCFH-DA (Figure 5A). The control cells showed only weak green fluorescence, indicating low ROS levels. The blank micelles (M)-treated group exhibited markedly enhanced green fluorescence intensity in MCF-7, HepG2, and 4T1 cells, indicating that blank micelles (M) could effectively elevate intracellular ROS levels. Combined with the results from the ROS response experiment, it is hypothesized that blank micelles (M) undergo ROS-responsive cleavage to release cinnamaldehyde, which further amplifies ROS generation.

#### 2.4.3. Release of Drug-Loaded Micelles (M@PPT) In Vitro

In the in vitro release assay (Figure 5D), free PPT exhibited a cumulative release rate of 97.23% within 24 h in PBS (pH 7.4), while the drug-loaded micelles (M@PPT) showed a significantly lower cumulative release rate of 64.78% under identical conditions. Notably, M@PPT demonstrated a minimal initial burst release, with only 17.12% of PPT released within 0.5 h, well below the 40% threshold typically associated with burst release behavior. These findings collectively demonstrate the sustained-release capability of M@PPT. Furthermore, in PBS containing 10 mM H_2_O_2_, the cumulative release of PPT from M@PPT reached 77.76% at 24 h, representing a statistically significant enhancement compared to the H_2_O_2_-free M@PPT group. The experimental data confirm the ROS-responsive release profile of M@PPT, as the micellar structure undergoes oxidative cleavage-triggered disintegration in the presence of 10 mmol/L H_2_O_2_, thereby accelerating PPT release.

The experimental results demonstrate that, at high ROS concentrations, the blank micelles (M) undergo responsive cleavage and release cinnamaldehyde, which in turn further elevates intracellular ROS levels. This increased ROS level accelerates micellar disintegration, thereby establishing a self-amplifying positive feedback loop. This mechanism not only continuously promotes micelles dissociation, but also significantly enhances the release efficiency of the drug PPT.

### 2.5. Preliminary Exploration of the In Vitro Antitumor Effect of the Drug-Loaded Micelles (M@PPT) and the Underlying Mechanism

#### 2.5.1. In Vitro Cytotoxicity of the Drug-Loaded Micelles (M@PPT)

**Figure 6:**
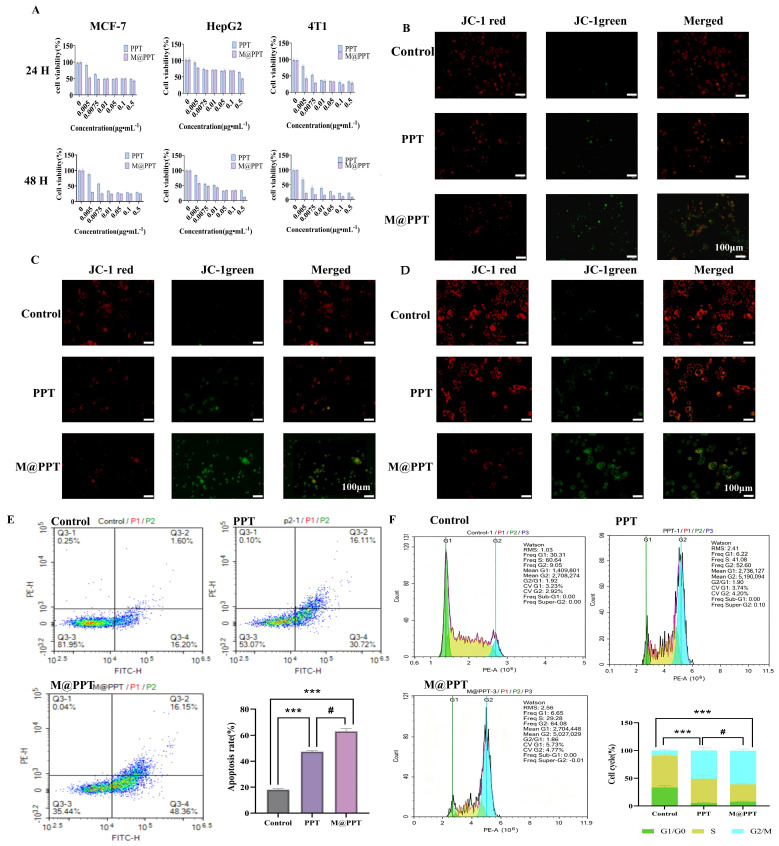
(**A**) Cytotoxicity of drug-carrying micelles (M@PPT) and PPT toward MCF-7, HepG2, and 4T1 cells in vitro. The effect of drug-loaded micelles (M@PPT) on mitochondrial membrane potential in MCF-7 (**B**), HepG2 (**C**), and 4T1 (**D**) cells was investigated using JC-1. (**E**) Effects of the drug-loaded micelles (M@PPT) and PPT on apoptosis of 4T1 cells. (**F**) Effects of the drug-loaded micelles (M@PPT) and PPT on the cell cycle in 4T1 cells. *** *p* < 0.001 is considered as extremely significant difference; # *p* < 0.05 represents a significant difference compared with the PPT group.

In the CCK-8 assay (Figure 6A), both drug-loaded micelles (M@PPT) and free PPT inhibited the proliferation of three tumor cell lines (MCF-7, 4T1, and HepG2), but the inhibitory effects on the growth of MCF-7 and 4T1 cells were more significant. In addition, at 48 h, the drug-loaded micelles (M@PPT) showed better growth inhibition of MCF-7 and 4T1 cells than free PPT.

#### 2.5.2. Effect of Drug-Loaded Micelles (M@PPT) on Mitochondrial Membrane Potential

Mitochondrial membrane potential was monitored using a JC-1 probe obtained from Beyotime Biotech Inc. (Shanghai, China). A decrease in mitochondrial membrane potential (ΔΨm depolarization) serves as a critical marker of early apoptosis [35]. In normal cells, JC-1 exhibits red fluorescence, whereas early apoptotic cells display green fluorescence due to ΔΨm depolarization. The control group showed only red fluorescence. Both the PPT- and M@PPT-treated groups exhibited significantly enhanced green fluorescence, with the M@PPT group demonstrating higher fluorescence intensity than the PPT group (Figure 6B–D). These results indicate that PPT and M@PPT can induce tumor cell apoptosis, and M@PPT exhibits stronger efficacy.

#### 2.5.3. Effects of the Drug-Loaded Micelles (M@PPT) on Apoptosis and the Cell Cycle in 4T1 Cells

In the cell apoptosis experiment (Figure 6E), after the drug-loaded micelles (M@PPT) acted on 4T1 cells for 2 days, the cell apoptosis rate was 64.51%, while the corresponding cell apoptosis rate for PPT was 46.83%. These results indicated that both M@PPT and PPT significantly promoted apoptosis of 4T1 cells, and the drug-loaded micelles (M@PPT) had a strong effect.

In the cell cycle experiment (Figure 6F), compared with the control group, both the drug-loaded micelles (M@PPT) and the free PPT could arrest the cell cycle of 4T1 cells at the G2/M phase. The arresting effect of the drug-loaded micelles (M@PPT) was more significant, which can be attributed to the greater uptake by 4T1 cells of the drug-loaded micelles (M@PPT).

#### 2.5.4. Effects of Drug-Loaded Micelles (M@PPT) on Growth-Related Proteins

**Figure 7:**
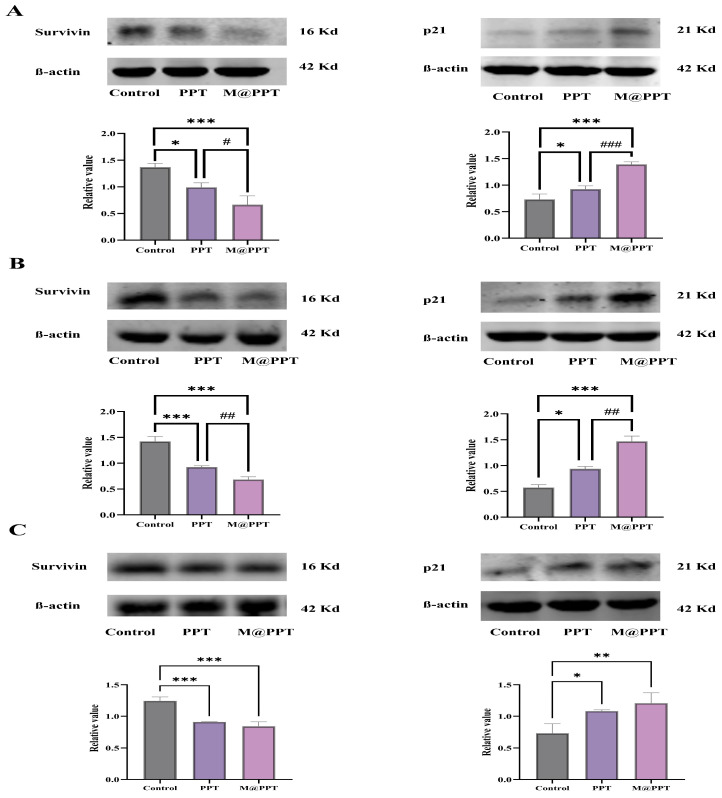
Effect of drug-loaded micelles (M@PPT) on Survivin and p21 expression in MCF-7 (**A**), HepG2 (**B**), and 4T1 (**C**) cells. * *p* < 0.05, ** *p* < 0.01 and *** *p* < 0.001 are considered as significant, highly and extremely significant differences, respectively; # *p* < 0.05, ## *p* < 0.01 and ### *p* < 0.001 represent significant, highly and extremely significant differences compared with the PPT group.

The drug-loaded micelles (M@PPT) downregulated the expression of the apoptosis-inhibitory protein Survivin and upregulated the expression of the cyclin-dependent kinase inhibitor p21 in MCF-7 (Figure 7A), HepG2 (Figure 7B), and 4T1 cells (Figure 7C). The experimental results indicate that the antitumor mechanism of the drug-loaded micelles (M@PPT) is related to downregulation of the expression level of the Survivin protein and upregulation of the expression level of the p21 protein.

### 2.6. In Vivo Antitumor Experiments Using Drug-Carrying Micelles (M@PPT)

#### 2.6.1. In Vivo Distribution of Micelles Loaded with DiI (M@DiI)

**Figure 8:**
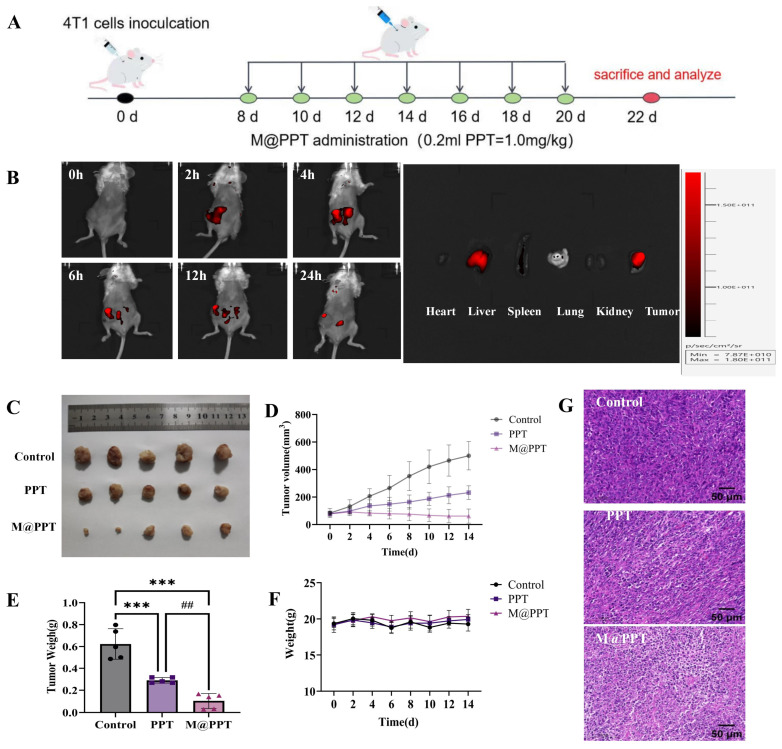
In vivo distribution of the DiI-loaded micelles (M@DiI) and in vivo antitumor experiment using the PPT-loaded micelles (M@PPT). (**A**) Schematic diagram of the treatment timeline in vivo. (**B**) After subcutaneous injection of M@Dil, whole-body fluorescence imaging of the mice was performed, followed by fluorescence imaging of various organs. (**C**). Photographs of mouse tumors dissected after death. (**D**) Tumor volume curves for the mice in each group. (**E**) Tumor weights at the end of the experimental period, *** *p* < 0.001 vs. Control, ## *p* < 0.01 vs. PPT. (**F**) Changes in the body weight of the mice in each group during treatment. (**G**) Tumor H&E staining in each group.

In the biodistribution study (Figure 8A), 1′-dioctadecyl-3,3,3′,3′-tetramethylindocarbocyanine perchlorate (DiI)-loaded micelles (M@DiI) accumulated in tumor tissues at 2 h post-injection, peaked at 4 h, and gradually decreased thereafter due to metabolic clearance. Fluorescence imaging of dissected organs (heart, spleen, lung, and kidney) revealed no detectable fluorescence, while significant fluorescence signals were observed exclusively in tumor tissues and liver organs, which indicated that the M@Dil micelles were enriched in tumor tissue and metabolized through the liver. Both ex vivo organ imaging and in vivo fluorescence tracking confirmed the preferential accumulation of M@DiI in tumors.

#### 2.6.2. Antitumor Effect and Biosafety Evaluation of Drug-Loaded Micelles (M@PPT) In Vivo

To evaluate the antitumor effect of drug-loaded micelles (M@PPT) in vivo, tumor-bearing mice were given the same dose of PPT and drug-loaded micelles (M@PPT) every 2 days for 14 days. Body weight and tumor volume were recorded every 2 days during the dosing cycle. During the 14-day administration cycle, the body weight of mice in the control group, PPT group, and drug-loaded micelle (M@PPT) group had no significant changes (Figure 8F). The tumor volume in the PPT group decreased compared with the control group. The tumor volume of the drug-loaded micelle (M@PPT) group showed a decreasing trend, indicating that the drug-loaded micelles (M@PPT) had a good tumor inhibition effect (Figure 8D). Dissected tumor tissues were photographed and weighed, revealing the highest tumor weight in the control group and the lowest in the M@PPT group, consistent with the tumor volume trends (Figure 8C,E).

H&E staining of tumor tissues showed high-density, disordered, and vigorously growing cells in the control group (Figure 8G). In contrast, cell growth in the PPT and M@PPT groups was slow. M@PPT-treated cells were sparse and exhibited shrinkage and nuclear fragmentation, consistent with the tumor volume changes. H&E staining of the tumor tissues showed that the cells in the control group had high density, disordered arrangement, and vigorous growth, while the cells in the PPT group and the drug-carrying micelle (M@PPT) group had slow growth. Cells in the drug-carrying micelle (M@PPT) group were sparse, and cell shrinkage and nuclear fragmentation were observed, which was consistent with the trends in tumor volume change.

The drug-loaded micelles (M@PPT) exhibit enhanced antitumor efficacy, primarily due to two factors. First, while free PPT undergoes rapid enzymatic degradation and systemic clearance from the circulation, M@PPT enables passive tumor targeting through the EPR effect, prolonging drug retention [36]. Second, the ROS-responsive drug release from M@PPT selectively increases the local drug concentration in tumor tissue, thereby improving therapeutic outcomes.

**Figure 9:**
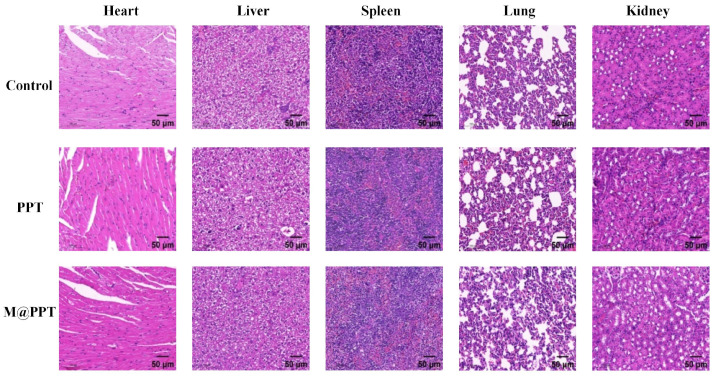
H&E staining of mouse organ sections for each group.

The results from the H&E staining of major organs (Figure 9) showed that the nuclei of liver and kidney sections in the PPT group were pyretic, broken, and blue-black, indicating that PPT had liver and kidney toxicity. However, there was no significant difference between the drug-carrying micelle (M@PPT group) and the control group. The above results indicate that drug-carrying micelles (M@PPT) have good biocompatibility and safety.

## 3. Discussion

Multiple orthogonal characterizations validate the successful assembly of uniform micelles: the amphiphilic mPEG-TCA-C_16_ copolymer was structurally confirmed via ^1^H-NMR and FT-IR spectroscopy, while the low critical micelle concentration (CMC), narrow unimodal dynamic light scattering (DLS) distribution, stable zeta potential, and sustained 5-day colloidal stability collectively demonstrate that ~26 nm micelles can stably exist in aqueous media; consistent cellular uptake, ROS-triggered drug release, and in vivo tumor accumulation further corroborate intact micellar structures. During TEM sample preparation, rapid solvent evaporation generates strong capillary force that collapses the hydrated mPEG outer shell and triggers irreversible aggregation of primary micelles into stacked clusters, where overlapping fused nanoparticles form continuous polymer films and obscure discrete spherical micelles. Based on the comprehensive characterization data obtained in aqueous solution, DLS measurements accurately reflect the intrinsic hydrodynamic diameter of hydrated micelles. Supplementary SEM imaging was not performed within the scope of the current study due to experimental limitations, and multi-dimensional electron microscopic characterization will be performed in our follow-up research to further validate the DLS-derived particle size results.

Thioacetal TCA exhibits stability under acidic and alkaline conditions but undergoes oxidative cleavage in the tumor microenvironment, which is characterized by elevated ROS [37], leading to cinnamaldehyde release. The liberated cinnamaldehyde exacerbates redox imbalance by depleting intracellular reducing agents, thereby amplifying ROS levels and establishing a positive feedback loop to accelerate TCA cleavage [38]. In our system, CA is covalently locked in the polymer backbone via thioacetal bonds; the α,β-unsaturated aldehyde active site is chemically shielded, precluding Michael addition with GSH thiols and eliminating the GSH-depleting capacity of conjugated CA. Under high ROS conditions in the tumor microenvironment, thioacetal linkages undergo selective oxidative cleavage, liberating free CA that then consumes GSH to disrupt redox homeostasis and amplify ROS, forming a self-amplifying cascade that accelerates micelle disassembly and drug release. Our DCFH-DA ROS imaging shows that blank CA-conjugated micelles significantly elevate intracellular ROS, while the increase in ROS levels in the control group is negligible, consistent with CA release and subsequent GSH depletion driving oxidative stress. Multiple independent studies confirm this mode of action: intact thioacetal-CA polymers do not alter cellular GSH levels, whereas ROS-released free CA robustly reduces GSH content and elevates ROS [39,40].

The ROS-responsive experiment showed that the blank micelles (M) exhibited an increase in particle size and release of cinnamaldehyde in response to stimulation with 10 mM H_2_O_2_. Moreover, under the Fenton-like conditions, the particle size distribution significantly broadened, and the release amount of cinnamaldehyde increased, confirming that the thioacetal TCA group has a specific response to hydroxyl radicals (•OH). Notably, thioketal TK-based nano-micelles demonstrated superior sensitivity to photosensitizer Ce6-generated singlet oxygen (^1^O_2_) [38]. This difference in the type of ROS response provides inspiration for the precise design of tumor microenvironment-activated nano drug delivery systems.

The in vitro release experiment demonstrated that the cumulative release rate of PPT from the drug-loaded micelles (M@PPT) reached 77.76% within 24 h in the buffer solution containing 10 mM H_2_O_2_, which was significantly higher than that of the group without H_2_O_2_ (64.78%), suggesting that ROS-triggered cleavage of the micelle structure can effectively promote drug release. The relatively high initial release rate may be related to the weak stability of drug encapsulation caused by the short hydrophobic chain segment of the polymer mPEG-TCA-C16 [32].

The in vivo experiment showed that the tumor volume of the group treated with the drug-loaded micelles (M@PPT) was significantly smaller than that of the group treated with free PPT. Our H&E micrographs of M@PPT-treated tumors show widespread nuclear pyknosis, karyorrhexis, cytoplasmic shrinkage, and focal necrotic areas compared with the free PPT and blank groups; these are classic morphological hallmarks of apoptotic and necrotic cell death in tumor tissue. These pathological changes, combined with our quantitative tumor volume/weight suppression and in vitro apoptotic data (Annexin V/PI, JC-1, Survivin downregulation), collectively support the synergistic antitumor activity of M@PPT micelles. Multiple published studies on cinnamaldehyde/ROS-responsive polymeric nano-therapies have validated that such H&E morphological alterations correlate well with TUNEL-positive apoptotic signals and enhanced therapeutic outcomes [39,41].

In this study, we successfully fabricated ROS-responsive drug-loaded micelles denoted M@PPT, which exhibited prominent antitumor efficacy and favorable biocompatibility both in vitro and in vivo, offering a facile strategy for the development of podophyllotoxin (PPT) formulations. Nevertheless, several limitations remain to be addressed for this micellar system. First, the compatibility between the micellar core and the encapsulated drug requires further improvement to suppress premature drug leakage under non-oxidative conditions. Subsequent structural modification can be implemented by introducing hydrophobic moieties with varied lipophilicity to adjust the hydrophilic–hydrophobic balance of the copolymer backbone [42]. Moreover, further systematic validation of the cascade mechanism of cinnamaldehyde (CA) responsive release and intracellular GSH depletion is another critical direction for follow-up investigations.

## 4. Materials and Methods

### 4.1. Materials

3-Mercaptopropionic acid, trifluoroacetic acid (TFA), cinnamaldehyde (CA), podophyllotoxin (PPT), hexadecanol, pyrene, and dichloromethane (water content ≤ 50 ppm) were purchased from Anergy Chemistry Co. 4-Dimethylaminopyridine (DMAP) and 1-(3-dimethylaminopropyl)-3-ethylcarbodiimide hydrochloride (EDCI) were obtained from Yuanye Bio-Technology Co., Ltd. Methoxy polyethylene glycol (mPEG2000) was acquired from TCI. 1,1′-Dioctadecyl-3,3,3′,3′-tetramethylindocarbocyanine perchlorate (DiI) and coumarin 6 were provided by Bide Pharmatech Co., Ltd. (Shanghai, China). Hoechst 33342, MTT, and CCK-8 were purchased from Biosharp (Hefei, China), Solarbio (Beijing, China), and GlpBio (Montclair, CA, USA), respectively. The mitochondrial membrane potential kit (JC-1), ROS detection kit, cell cycle and apoptosis kit, Survivin antibody, and p21 antibody were obtained from Beyotime (Shanghai, China). The β-actin antibody was provided by Servicebio (Wuhan, China). All other solvents were of analytical grade.

### 4.2. Synthesis of mPEG-TCA-C16

Dissolve 3-mercaptopropionic acid (2.335 g, 22 mmol) and CA (1.140 g, 10 mmol) in ethyl acetate; add TFA (0.228 g, 2.0 mmol) in an ice bath and react for 12 h while maintaining the ice-cold temperature [43]. After completion of the reaction, the mixture was filtered under vacuum, washed three times with water, washed three times with petroleum ether, and dried to yield TCA.

TCA (1.222 g, 3.75 mmol), EDCI (0.720 g, 3.75 mmol), and DMAP (0.305 g, 2.5 mmol) were dissolved in anhydrous methylene chloride and stirred for 10 min at room temperature. Then, hexadecanol (0.600 g, 2.5 mmol) was added, and the reaction was allowed to proceed at room temperature for 24 h. The reaction solution was washed three times with 0.01 mol/L diluted hydrochloric acid, deionized water, and saturated sodium chloride solution, respectively [27]. Subsequently, anhydrous magnesium sulfate was added to dry the organic phase, the desiccants were removed by filtration, and the filtrate was concentrated by spin evaporation and purified by silica gel column chromatography to obtain compound TCA-C16.

TCA-C16 (0.600 g, 1.1 mol), EDCI (0.422 g, 2.2 mmol), and DMAP (0.045 g, 0.37 mmol) were dissolved in anhydrous dichloromethane and stirred at room temperature for 10 min. Then, mPEG2000 (0.740 g, 0.37 mmol) was added, and the reaction was allowed to proceed at room temperature for 24 h. Remove the solvent, add an appropriate amount of isopropyl alcohol to dissolve in a water bath at 40 °C, precipitate at lower temperature, extract and filter, and recrystallize three times. After removing the solvent by rotary evaporation, the crude product was obtained by recrystallization from isopropyl alcohol three times. The crude product was then dialyzed (MWCO ≥ 2000 Da) against deionized water for 48 h, followed by lyophilization to yield mPEG-TCA-C16 [44].

### 4.3. Characterization

The ^1^H NMR spectra of TCA, TCA-C16, and mPEG-TCA-C16 were recorded using a Bruker AVANCE400 spectrometer (Bruker, Billerica, MA, USA). FT-IR spectra for TCA, TCA-C16, mPEG2000, and mPEG-TCA-C16 were obtained using a SHIMADZU Fourier (Shimadzu, Kyoto, Japan) transform infrared spectrometer from 4000 cm^−1^ to 400 cm^−1^.

### 4.4. Nanoparticle Preparation

The drug-loaded micelles (M@PPT) were prepared by the thin film dispersion method [45]. mPEG-TCA-C16 (20 mg) and PPT (2 mg) were dissolved in 5 mL methanol and evaporated in a water bath at 40 °C to form uniform films. Then, 10 mL PBS buffer solution was hydrated in a water bath at 55 °C for 10 min. M@PPT was obtained through a 0.22 μm filter membrane. The blank micelles (M) were prepared by the same method. Orthogonal experiments were carried out to optimize the feed ratio, organic solvent, hydration temperature, and hydration volume for M@PPT fabrication. The detailed experimental design, encapsulation efficiency (EE), and drug loading content (DL) results are listed in Table S1.

The *EE* and *DL* of drug-loaded micelles (M@PPT) were measured by the low speed centrifugation method [46]. First, 1 mL of drug-loaded micelles (M@PPT) was placed in a 1.5 mL centrifuge tube and centrifuged at 3000 rpm for 20 min. Then, 0.2 mL of the supernatant was diluted with the same amount of methanol, and 20 µL of the sample was injected into a high-performance liquid chromatography (HPLC) apparatus to convert the content of PPT. The *EE* and *DL* of the micelles are calculated according to the following formula:

$$\mathrm{EE}(\mathrm{wt}\;\%)=\frac{m_{\mathrm{encapsulation}}}{m_{\mathrm{feed}}}\times 100\%$$

$$\mathrm{DL}(\mathrm{wt}\;\%)=\frac{m_{\mathrm{encapsulation}}}{m_{\mathrm{feed}}+m_{\mathrm{carrier}}}\times 10$$

where m_feed_ represents the amount of PPT added during the preparation of drug-loaded micelles (M@PPT), and m_carrier_ represents the amount of mPEG-TCA-C16 added during the preparation of drug-loaded micelles (M@PPT).

### 4.5. Size, Zeta Potential, and Stability of Micelles

The particle size and *zeta* potential of blank micelles (M) and drug-loaded micelles (M@PPT) were determined by dynamic light scattering (DLS) (NaNoZS90, Malvern, Almelo, The Netherlands). The appearance of the micelles was characterized by TEM (JEM-2100, ZEISS, Tokyo, Japan). The particle size distribution of blank micelles (M) and drug-loaded micelles (M@PPT; 1 mg/mL) was monitored by DLS for 5 days at room temperature to assess colloidal stability.

### 4.6. ROS-Responsive Assay

Blank micelles (M) were incubated with PBS buffer (pH 7.4), PBS buffer (pH 7.4) containing 10 mM H_2_O_2_, or Fenton’s reagent (10 mM H_2_O_2_ + 100 μM Cu^2+^) [47] at room temperature for 12 h, and the changes in particle size and distribution were measured by DLS. The incubation solution was added with an equal volume of methanol, and the mixture was sonicated for 10 min, filtered through 0.22 μm membrane, and injected into an HPLC apparatus to qualitatively analyze the release of CA.

### 4.7. Hemolytic and Safety Experiments for Blank Micelles (M)

Rabbit heart blood was washed and centrifuged with normal saline three times, and then the red blood cells were diluted with normal saline to obtain a 4% red blood cell suspension. The blank micelle (M) solution was mixed with the red blood cell suspension to obtain 10–100 μg/mL micelle solutions, using water as the positive control and normal saline as the negative control, followed by incubation at 37 °C for 3 h. After centrifugation to observe hemolysis, the supernatant was collected to measure absorbance at 540 nm, with subsequent conversion to calculate hemolysis ratio (%).

The cytotoxicity of blank micelles (M) to human umbilical vein endothelial cells (HUVEC), human breast cancer cells (MCF-7), human liver cancer cells (HepG2), and mouse breast cancer cells (4T1) was determined by MTT assay. The cells were seeded in 96-well plates at a density of 4 × 10^3^ cells/well and cultured overnight, after which 100 µL blank micelle (M) solution diluted with complete medium at different concentrations was added to each well. After 48 h of incubation, 20 µL of MTT solution (5 mg/mL) was added to each well, and the cells were cultured for another 2 h. Add 100 µL of DMSO to each well and shake for 10 min to dissolve the formazan crystals. The optical density (OD) value of each well was detected at 492 nm using a microplate reader (Varioskan LUX, Thermo Fisher Scientific, Waltham, MA, USA) to calculate the cell survival rate (%).

### 4.8. CMC of Blank Micelles (M)

The critical micelle concentration (CMC) of blank micelles (M) was determined by fluorescence spectra (RF-6000, Shimadzu, Kyoto, Japan) using pyrene as a probe [48]. The solution concentrations of blank micelles (M) ranged from 5 × 10^−4^ to 1 mg/mL, with a final pyrene concentration of 1 × 10^−7^ mol/L. The solution was sonicated at room temperature for 30 min and then equilibrated in the dark for 1 day. The fluorescence emission wavelength was fixed at 373 nm, the excitation spectrum of 300~360 nm was tested, and the slit width was 5 nm [49]. Plot the ratio of the excited fluorescence intensities at 334 nm and 336 nm against the logarithm of various concentrations to estimate the CMC.

### 4.9. Cellular Uptake

MCF-7, HepG2, and 4T1 cells (20,000 cells/well) were seeded in a 24-well plate. After 24 h of culture, Cou6 solution and M@Cou6 solution (prepared according to the protocol in Section 2.4) were added, followed by diluted Hoechst 33342 live-cell staining solution at 2 h and 4 h. After incubation for 20 min, the samples were washed three times with PBS and imaged using a fluorescence microscope (NOVEL NIB610FL, Novel, Nanjing, China).

### 4.10. Release of Drug-Loaded Micelles (M@PPT) In Vitro

The dialysis method was used to determine the release of drug-loaded micelles (M@PPT) and PPT [50]. Briefly, 3 mL of drug-loaded micelle (M@PPT) solution (2 mg/mL) or free PPT was loaded into dialysis bags (MW = 1000 Da) and immersed in 57 mL of phosphate buffer (pH 7.4) containing 1.0% Tween 80. The M@PPT group was further divided into two groups, one with 10 mmol/L H_2_O_2_ and the other without H_2_O_2_ as the control group. All dialysis bags were incubated at 37 °C in a shaker at 100 rpm. At 0.5, 1, 3, 6, 9, 12, and 24 h, 1 mL of the release medium was collected and supplemented with 1 mL to keep the total volume constant, and the release medium was analyzed by HPLC to measure the PPT content.

### 4.11. Intracellular ROS Generation

A DCFH-DA assay kit [45] was utilized to investigate the effect of blank micelles (M) on intracellular ROS levels in MCF-7, HepG2, and 4T1 cells. Cells (2 × 10^4^ cells/well) were seeded in a 24-well plate and cultured overnight, followed by treatment with blank micelle (M) solution for 48 h. After incubation for 48 h, the cells were incubated with blank medium containing DCFH-DA for 30 min, and the fluorescence of the cells was observed using a fluorescence microscope (NOVEL NIB610FL, Novel, Nanjing, China).

### 4.12. In Vitro Cytotoxicity

The in vitro cytotoxicity of drug-loaded micelles (M@PPT) and free PPT to MCF-7, HepG2, and 4T1 cells was investigated by the CCK-8 method [51]. First, 96-well plates were inoculated with 4 × 10^3^ cells per well. After overnight incubation, 100 µL of complete medium containing 0, 0.005, 0.0075, 0.01, 0.05, 0.1, or 0.5 μg/mL of free PPT or drug-loaded micelles (M@PPT; based on PPT content) was added to each well. After 24 or 48 h, the old medium was removed, and 100 µL 10% CCK-8 solution was added to each well. After 2 h of continuous culture, the OD value of each well was detected at 450 nm using an enzyme-labeler, and the survival rate (%) was calculated.

### 4.13. JC-1 Assay

The effects of drug-loaded micelles (M@PPT) and PPT on the mitochondrial membrane potential of MCF-7, HepG2, and 4T1 cells were investigated with a JC-1 kit [51]. Cells were seeded in 24-well plates at 2 × 10^4^ cells per well and cultured overnight; PPT or drug-loaded micelles (M@PPT) were added to each well, and the cells were cultured for another 24 h. Each well was incubated with 0.5 mL JC-1 dye solution for 30 min, then washed with JC-1 dye buffer there times, and then observed under a fluorescence microscope (NOVEL NIB610FL, Novel, Nanjing, China).

### 4.14. Cell Cycle and Apoptosis

4T1 cells were seeded into six-well plates and treated with PPT and drug-loaded micelles (M@PPT) for 24 h. After treatment, the cells were collected and fixed overnight with cold 70% ethanol at 4 °C. Then, the fixed cells were washed twice with PBS and incubated for 30 min at 37 °C away from light with PI staining reagent. Then, the PI fluorescence intensity was measured by flow cytometry (NovoCyte, Agilent, Santa Clara, CA, USA), and the cell cycle profile was fitted.

4T1 cells were seeded into six-well plates and cultured for 24 h, then treated with fresh medium containing PPT or M@PPT and further cultured for 24 h. After 24 h, the cells were collected and stained with Annexin V-FITC and PI and assessed by flow cytometry (NovoCyte, Agilent, Santa Clara, CA, USA).

### 4.15. Western Blot

4T1 cells were incubated with PPT or M@PPT for 48 h, and then protein samples were extracted from the cells with RIPA lysis buffer. The protein samples were separated by 12% SDS-PAGE and transferred to nitrocellulose (NC) membranes. The membranes were blocked with 5% skim milk powder for 2 h at room temperature and then incubated overnight at 4 °C with primary antibodies against Survivin (1:1000), p21 (1:500), and β-actin (1:1000) and subsequently incubated with a fluorescent secondary antibody at room temperature for 1.5 h. Finally, the protein bands were detected using an Odyssey DLx Imaging System (Odyssey DLx, LI-COR, Lincoln, NE, USA), and signal intensities were quantified using Quantity One^®^ software, Version 4.6.6.

### 4.16. Establishment of the Tumor Model

Balb/c female mice aged 5–6 weeks (18–20 g) were purchased from Liaoning Changsheng Biotechnology Co., Ltd. (Benxi, China). All procedures were approved by the Institutional Animal Care and Use Committee (IACUC) of Harbin Medical University (License No. IRB3119724).

A 4T1 cell suspension (5 × 10^7^ cells/mL) was injected subcutaneously into the right leg of Balb/c female mice at an injection volume of 200 µL per mouse. When the tumor volume grew to 80~100 mm^3^, the mouse breast cancer heterotopic transplantation tumor model was successfully established.

### 4.17. Distribution In Vivo

The tumor-bearing mice were intravenously injected via the tail vein with 200 µL of M@DiI micelles (prepared as per Section 2.4), and the fluorescence distribution in vivo was imaged using a small animal fluorescence imaging system at 0, 2, 4, 6, 12, and 24 h. Twenty-four hours after the injection, sacrifice the mice by cervical dislocation, dissect and remove the tumor tissues as well as the heart, liver, spleen, lungs, and kidney, and capture the fluorescence images of the distribution of M@DiI micelles in the tumors and organs.

### 4.18. In Vivo Antitumor Efficacy and Safety Evaluation

When the tumor volume reached 80–100 mm^3^, tumor-bearing mice were randomly divided into three groups (n = 5 per group) and injected subcutaneously with 0.2 mL every other day for 14 days (seven doses total). The control group received normal saline, the PPT group received PPT at 1.0 mg/kg, and the M@PPT group was treated with M@PPT containing 1.0 mg/kg PPT equivalent. The mice were weighed, and the length and width of the tumors were measured the day after each administration. The tumor volume is calculated as V = (ab^2^)/2.

At the end of the experimental period, the mice were killed by cervical dislocation, and the tumors, heart, liver, spleen, lungs, and kidneys were collected, washed with PBS, and dried, and photographs were taken. H&E staining was performed of the tumor tissues and major organs to study the therapeutic effect and potential organ toxicity of M@PPT.

### 4.19. Statistical Analysis

The statistical experiments were repeated three times, and the results were expressed as mean ± SD. Statistical processing was performed using the one-way ANOVA method in GraphPad Prism 9.0 software. *p* < 0.05 is considered a statistical difference, *p* < 0.01 indicates a significant statistical difference, and *p* < 0.001 indicates an extremely significant statistical difference (*, *p* < 0.05; **, *p* < 0.01; ***, *p* < 0.001).

## 5. Conclusions

In this study, drug-loaded micelles (M@PPT) with good ROS responsiveness, high quality, and excellent biocompatibility were successfully developed. In both in vitro and in vivo tumor experiments, M@PPT demonstrated good antitumor activity. Their antitumor mechanism is achieved by downregulating the expression of the apoptosis-inhibiting protein Survivin and upregulating the expression of the p21 protein. This drug delivery system can respond to endogenous ROS and release PPT and CA. Moreover, based on the effect of CA on increasing the ROS level, a positive feedback mechanism is formed to promote the release of PPT, effectively improving the targeting and therapeutic effect of PPT and providing a new strategy for the formulation of PPT.

## Data Availability

The original contributions presented in this study are included in the article/Supplementary Materials. Further inquiries can be directed to the corresponding author.

## Supplementary Materials

The following supporting information can be downloaded at: https://www.mdpi.com/article/10.3390/ijms27156546/s1.
