# Supplementary material for: ROS-Responsive Micelles Loaded with Podophyllotoxin Inhibit Tumor Growth via ROS Self-Amplification and Regulation of Survivin and p21 Expression
Source: Int J Mol Sci. 2026 Jul 23;27(15):6546. doi: 10.3390/ijms27156546 (PMC13466196; doi:10.3390/ijms27156546)
Supplement: Supplementary file 1 [file ijms-27-06546-s001.zip › ijms-4393464-supplementary.pdf]

Synthetic route of TCA, TCA-C16, and mPEG-TCA-C16

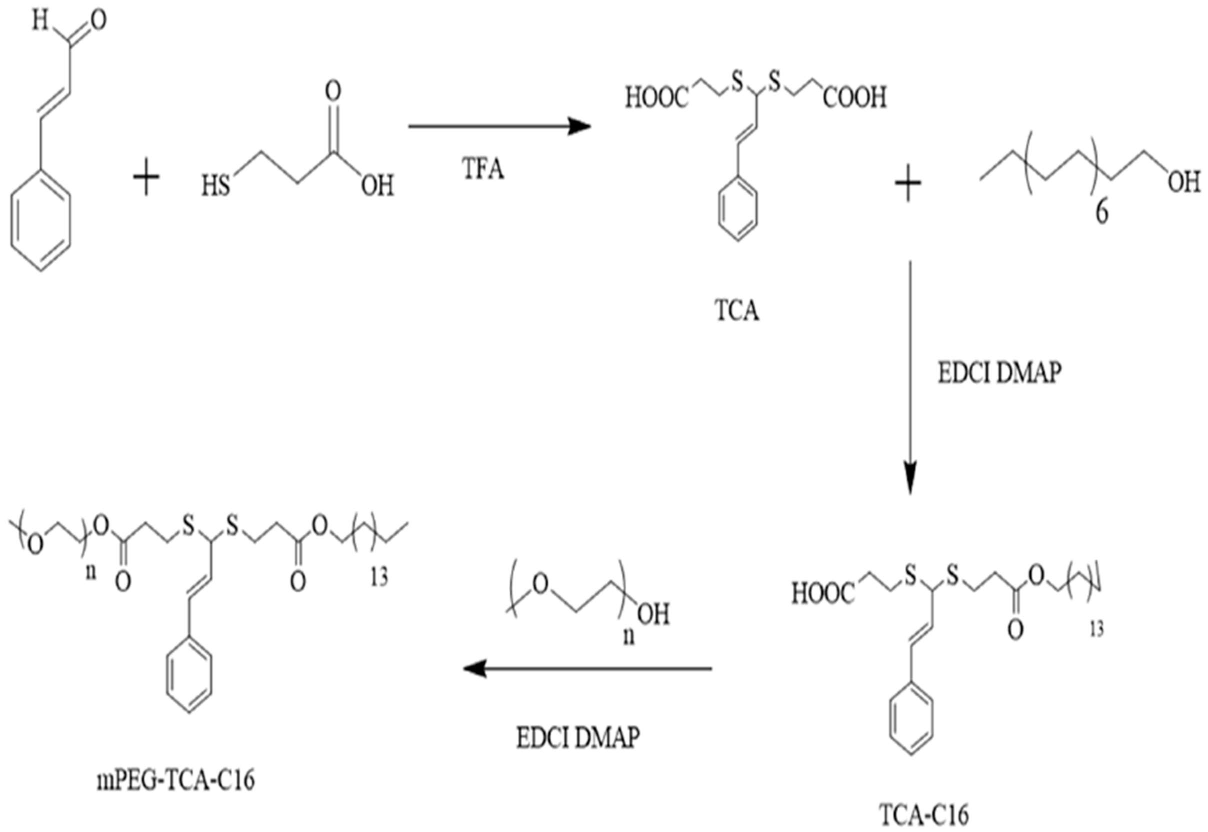

Figure S1. Synthesis scheme of mPEG-TCA-C16.

Determination of PPT Content by HPLC and Specificity Investigation

Under isocratic elution conditions (V methanol: V water = 55: 45), injection of free PPT and mPEG-TCA-C16 was used to assess the specificity of PPT. As shown in Figure S2, the retention time of PPT is approximately 10.5 minutes, with a single, symmetrical peak. Under these conditions, no sample peak for mPEG-TCA-C16 was observed. This indicates that mPEG-TCA-C16 does not interfere with the quantitative analysis of PPT, and that the specificity of PPT under isocratic elution conditions (V methanol: V water = 55: 45) is excellent.

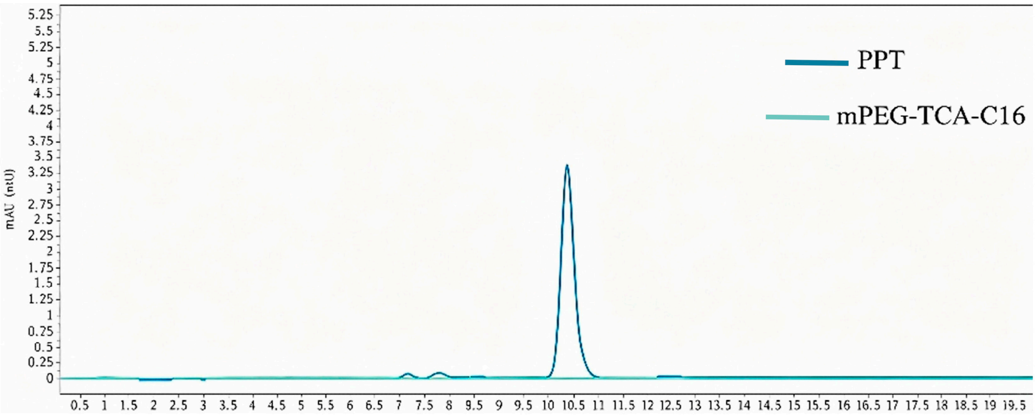

Figure S2. Specificity test for PPT content determination.

Quantitative Analysis of Cellular Uptake of Coumarin 6 (Cou6)-loaded blank micelles (M) (M@Cou6)

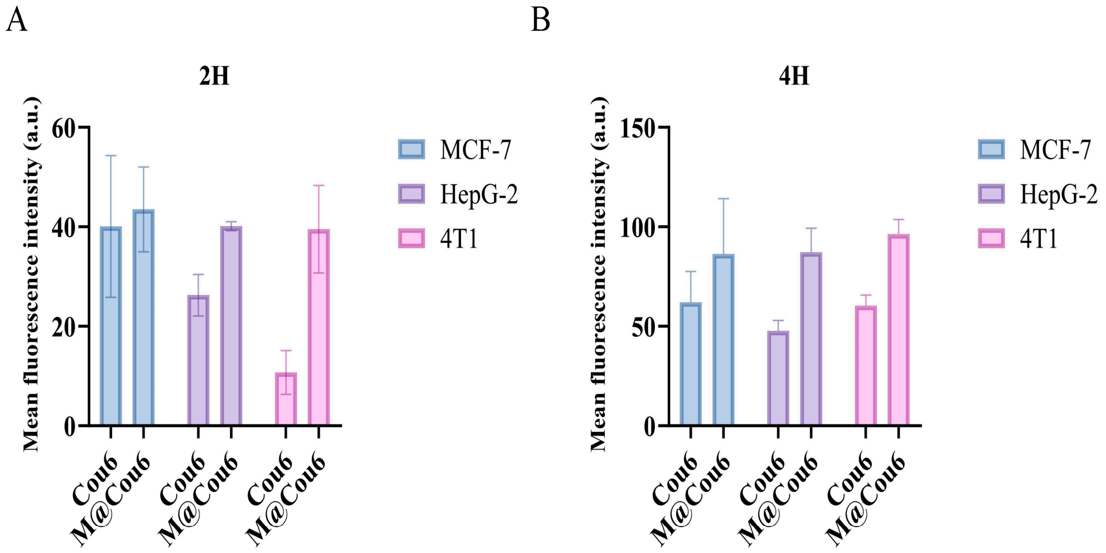

Figure S3. Quantitative cellular uptake analysis of Coumarin 6 (Cou6)-loaded blank micelles (M) (M@Cou6). (A) Mean fluorescence intensity of tumor cells after 2 h of incubation; (B) mean fluorescence intensity of tumor cells after 4 h of incubation.

Orthogonal Experimental Optimization of the Formulation and Process for Preparing Drug-Loaded Micelles (M@PPT)

Table S1. The orthogonal design and *EE* and *DL* results.

	Feed ratio	Solvent	Hydration temperature (℃)	Hydration volume (mL)	*EE* (%)	*DL* (%)
1	5:1	Dichloromethane	35	5	40.32	6.72
2	5:1	Anhydrous ethanol	55	10	94.39	15.73
3	5:1	Methanol	45	20	89.64	14.94
4	10:1	Dichloromethane	55	20	96.94	8.81
5	10:1	Anhydrous ethanol	45	5	98.44	8.95
6	10:1	Methanol	35	10	96.99	9.21
7	20:1	Dichloromethane	45	10	62.61	2.98
8	20:1	Anhydrous ethanol	35	20	71.02	3.38
9	20:1	Methanol	55	5	92.24	4.39
EE						
k_1	74.96	66.62	69.44	77.00		
k_2	97.45	87.95	83.56	84.66		
k_3	75.29	92.92	94.52	85.87		
R	22.49	26.72	25.08	8.87		
Solvent > Hydration Temperature > Feed Ratio > Hydration Volume. Optimal conditions: 10:1 methanol, 55 °C, 20 mL.						
DL						
k_1	12.46	6.17	6.43	6.69		
k_2	8.99	9.35	8.96	9.30		
k_3	3.58	9.51	9.64	9.04		
R	8.88	3.18	3.21	2.61		
Feed ratio > Hydration temperature > Solvent > Hydration volume. Optimal conditions: 5:1 methanol, 55°C, 10 mL.						
